# Supplementary material for: A corticostriatal deficit promotes temporal distortion of automatic action in ageing
Source: eLife. 2017 Oct 23;6:e29908. doi: 10.7554/eLife.29908 (PMC5677368; doi:10.7554/eLife.29908)
Supplement: Transparent reporting form [file elife-29908-transrepform.pdf]

eLIFE
1st Floor
24 Hills Road
Cambridge CB2 1JP, UK
P 01223 855340
W elifesciences.org
T @elife

eLife's transparent reporting form

We encourage authors to provide detailed information *within their submission* to facilitate the interpretation and replication of experiments. Authors can upload supporting documentation to indicate the use of appropriate reporting guidelines for health-related research (see EQUATOR Network), life science research (see the BioSharing Information Resource), or the ARRIVE guidelines for reporting work involving animal research. Where applicable, authors should refer to any relevant reporting standards documents in this form.

If you have any questions, please consult our Journal Policies and/or contact us: editorial@elifesciences.org.

Sample-size estimation

- You should state whether an appropriate sample size was computed when the study was being designed
- You should state the statistical method of sample size computation and any required assumptions
- If no explicit power analysis was used, you should describe how you decided what sample (replicate) size (number) to use

Please outline where this information can be found within the submission (e.g., sections or figure legends), or explain why this information doesn't apply to your submission:

This study compares performance between young and aged mouse cohorts. The central result involves within-between subjects interaction of lever press (LP) performance across the factors age (between subjects) and training day (within-subjects). To determine which sample size would allow statistical reporting of LP rate differences in this interaction with sufficient power, we ran a first pilot study in which 8 animals were used in each group (8 young and 8 aged mice, 15 days of training). This provided accurate effect size measures (i.e. partial η^2=0.217) and achieved a power of 0.85 (see G*Power report below; α level = 0.05; sphericity assumed). Based on this, we considered that a sample size of 8 offered an excellent statistical power/animal use ratio to reject the null hypotheses in all experiments conducted in this study (Cohen J. (1988). Statistical Power Analysis for the Behavioral Sciences, Hillsdale, NJ). In the instances where this sample size was significantly compromised (subject exclusions typically due to health issues in aged subjects), an additional power analysis was conducted to ensure the statistical validity of the results (see Replicates section below).

F tests - ANOVA: Repeated measures, within-between interaction

Analysis:	Post hoc: Compute achieved power		
Input:	Effect size f(U)	=	0.5264401 (partial η^2=0.217)
	α err prob	=	0.05
	Total sample size	=	8
	Number of groups	=	2
	Number of measurements	=	15
	Nonsphericity correction ε	=	1 (sphericity assumed)
Output:	Noncentrality parameter λ	=	23.2796910
	Critical F	=	1.8112970
	Numerator df	=	14.0000000
	Denominator df	=	84.0000000
	Power (1-β err prob)	=	0.8498564

eLife Sciences Publications, Ltd is a limited liability non-profit non-stock corporation incorporated in the State of Delaware, USA, with company number 5030732, and is registered in the UK with company number FC030576 and branch number BR015634 at the address 1st Floor, 24 Hills Road, Cambridge CB2 1JP | August 2014

Replicates

- You should report how often each experiment was performed
- You should include a definition of biological versus technical replication
- The data obtained should be provided and sufficient information should be provided to indicate the number of independent biological and/or technical replicates
- If you encountered any outliers, you should describe how these were handled
- Criteria for exclusion/inclusion of data should be clearly stated
- High-throughput sequence data should be uploaded before submission, with a private link for reviewers provided (these are available from both GEO and ArrayExpress)

Please outline where this information can be found within the submission (e.g., sections or figure legends), or explain why this information doesn't apply to your submission:

The details of each experiment are found in the corresponding SourceData files ('\Experiment summary' and '\Data type and description' captions). These contain all the information on the number of independent biological replicates used in each experiment, as well as the criteria for exclusion of data points/subjects. SourceData files are referred to in the corresponding panels of each figure and figure supplement (legend).

Statistical reporting

- Statistical analysis methods should be described and justified
- Raw data should be presented in figures whenever informative to do so (typically when N per group is less than 10)
- For each experiment, you should identify the statistical tests used, exact values of N, definitions of center, methods of multiple test correction, and dispersion and precision measures (e.g., mean, median, SD, SEM, confidence intervals; and, for the major substantive results, a measure of effect size (e.g., Pearson's r, Cohen's d)
- Report exact p-values wherever possible alongside the summary statistics and 95% confidence intervals. These should be reported for all key questions and not only when the p-value is less than 0.05.

Please outline where this information can be found within the submission (e.g., sections or figure legends), or explain why this information doesn't apply to your submission:

Full statistical reports (StatReport files, outputted in IBM SPSS Statistics 24 software) were obtained for each individual result, and are referred to in each corresponding figure and figure supplement panels in the legend. These reports include a 'Tabular Results' section with detailed information on the statistical test applied, factor definition, sample size, homogeneity and sphericity tests with appropriate corrections, descriptive statistics, confidence intervals, alpha levels, exact p values, between subjects effects, within-subjects effects and, if appropriate, bootstrapping, effect sizes and power. The reports also include a 'Narrative Results" section which is similar to the statistical results present in the Results section of the manuscript.

(For large datasets, or papers with a very large number of statistical tests, you may upload a single table file with tests, Ns, etc., with reference to sections in the manuscript.)

Group allocation

- Indicate how samples were allocated into experimental groups (in the case of clinical studies, please specify allocation to treatment method); if randomization was used, please also state if restricted randomization was applied
- Indicate if masking was used during group allocation, data collection and/or data analysis

Please outline where this information can be found within the submission (e.g., sections or figure legends), or explain why this information doesn't apply to your submission:

All details on counterbalancing and group allocation are found in the Materials and Methods section of the manuscript, as well as in the "Experimental summary" captions in each SourceData file.

Additional data files ("source data")

- We encourage you to upload relevant additional data files, such as numerical data that are represented as a graph in a figure, or as a summary table
- Where provided, these should be in the most useful format, and they can be uploaded as "Source data" files linked to a main figure or table
- Include model definition files including the full list of parameters used
- Include code used for data analysis (e.g., R, MatLab)
- Avoid stating that data files are "available upon request"

Please indicate the figures or tables for which source data files have been provided:

Source data files and Statistics reports are provided for all results in the manuscript. This includes SourceData files 1 to 6 and StatsReport files 1 to 22.
